# The impact of the COVID-19 pandemic on the health and working conditions of nurses and its implications for policies: a cross-sectional study in Slovakia

**DOI:** 10.1186/s12912-023-01356-z

**Published:** 2023-05-29

**Authors:** Silvia Putekova, Jana Martinkova, Alena Urickova, Lukas Kober, Stanislava Reichertova, Dominika Plancikova, Marek Majdan

**Affiliations:** 1 Faculty of Health Sciences and Social Work, Department of Nursing, Trnava University, Trnava, Slovakia; 2 Faculty of Health, Department of Nursing, Catholic University, Ruzomberok, Slovakia; 3 Slovak Chamber of Nurses and Midwives, Bratislava, Slovakia; 4 Department of Paramedic Science, Medical Diagnostic Studies and Public Health, Faculty of Health Care Studies, University of West Bohemia, Pilsen, Czech Republic; 5 Institute for Global Health and Epidemiology, Department of Public Health, Faculty of Health Sciences and Social Work, Trnava University, Hornopotocna 23, 91843 Trnava, Slovakia

**Keywords:** Healthcare Workers, COVID-19, Health Policies, Health Impact, Nursing Personnel

## Abstract

**Background:**

Increased workload and of the health workforce (HW) strained the capacity to maintain essential health services (EHS) during the Coronavirus Disease 2019 (COVID-19) pandemic, while putting them at increased risk of COVID-19 and other consequences to their health. The aim of this study was to assess the impact of COVID-19 on the health, wellbeing, and working conditions of nurses in Slovakia and to identify gaps in policies to be addressed to increase preparedness of the HW for future emergencies.

**Methods:**

A nation-wide cross-sectional study was conducted among nurses during November–December 2021, referring to the period of January 2021 to November 2021. To assess the differences between impact on HW on various levels of care, respondents were grouped by type of facility: hospital-COVID-19 wards; Hospital–non-covid ward; Outpatient or ER; Other care facilities.

**Results:**

1170 nurses participated, about 1/3 of them tested positive for COVID-19 by November 2021, mostly developing mild disease. Almost 2/3 reported long-covid symptoms and about 13% reported that they do not plan to get vaccinated against COVID-19. The median of the score of the impact of workload on health was 2.8 (56% of the maximum 5), the median score of mental health-wellbeing was 1.9 (63% of a maximum of 3). The studied impacts in all domains were highest in nurses working in COVID-19 hospital wards. Significant disruptions of health care were reported, with relatively high use of telemedicine to mitigate them. Overall, about 70% of the respondents thought of leaving their job, mostly due to working stress or inadequate pay.

**Conclusions:**

Our study showed that the COVID-19 pandemic poses a substantial burden on the health, wellbeing and working conditions of nurses in Slovakia and that a large proportion of nurses considered leaving their jobs because of work overload or low salaries. Human resource strategies should be adopted to attract, retain and continuously invest in HW development including in emergency preparedness and response. Such an approach may improve the resilience and preparedness of the health system in Slovakia for future emergencies.

**Supplementary Information:**

The online version contains supplementary material available at 10.1186/s12912-023-01356-z.

## Introduction

By April 2023, over 275 million cases of COVID-19 have been confirmed within the World Health Organization (WHO) European region, resulting in over 2.8 million deaths [1]. Besides these direct impacts on health of the population, the COVID-19 pandemic put a major burden on health systems of countries: the second round of the National pulse survey on continuity of essential health services (EHS) during the COVID-19 pandemic estimated that in nearly all responding countries (94%) at least some disruption of EHS was observed [2].

The health workforce (HW) is a key component for an effective response to the COVID-19 pandemic and for maintaining EHS; indeed over 66% of countries reported that HW-related disruptions represent the most common causes of disruptions of EHS [2]. The pandemic impacted the availability and capacity of the HW to deliver essential services and meet surge needs, the main challenges including lack of adequate personal protective equipment (PPE) and other essential equipment (such as laboratory equipment, ventilators of medicines used in intensive care), infection and quarantine, social discrimination and attacks, dual responsibility to care for friends and family members, and redistribution of staff to treat COVID-19 patients [3, 4].

The combination of the increased workload and a the reduced number of HW is expected to severely strain the capacity of health systems to maintain EHS, which should be offset through a combination of strategies—including recruitment, repurposing within the limits of training and skills, redistributing roles among the HW, while at the same time keeping the HW safe and providing mental health and psychosocial support [4]. The WHO developed a comprehensive guidance to design, manage and preserve the workforce necessary to manage the COVID-19 pandemic and maintain EHS, which covers the following domains: supporting and protecting HW (E.g., infection prevention and control, provision of PPE, incentives, mental health support), strengthening and optimizing HW teams (E.g., optimizing roles, training), increasing capacity and strategic HW deployment (E.g., improving HW availability through hiring and redeployment, rationalizing HW distribution), and health system human resources strengthening (E.g., improving HW information systems, planning of HW needs, strengthening governance and intersectoral collaboration mechanisms) [3].

At the same time, the HW is at increased risk of COVID-19 and other consequences to their health stemming from increased workload and responsibilities [5]. The risk of infection with COVID-19 in health workers is substantially higher, compared to the general population. A systematic review concluded that besides those in close contacts with COVID-19 infected persons, high-risk HW had significantly higher seroprevalence, compared to the general population [6]. The WHO estimated that between January 2020 and May 2021, 115,500 health workers died due to COVID-19, and this figure likely underestimates the true death toll [7]. Health workers are known to be at risk for anxiety, depression, burnout, insomnia, moral distress, and post-traumatic stress disorder. Under usual working conditions, severe burnout syndrome affects as many as 33% of critical care nurses and up to 45% of critical care physicians. Extrinsic organizational risk factors (e.g., increased work demands, little control over the work environment), and the trauma of caring for critically ill patients were heightened by the COVID-19 pandemic and represent important exacerbating factors for poor mental health among the HW [8]. A systematic review including a population of 90,000 nurses reported that over one third of them experienced stress, sleep disturbances and increased mood and anxiety symptoms due to the COVID-19 pandemic, which was considerably higher than findings during smaller-scale pandemics like Severe Acute Respiratory Syndrome (SARS), or compared to the general population at the same time period [9].

The above findings warrant for detailed monitoring of the health impacts of COVID-19 on the HW in order to elucidate their causes and dynamics and to provide evidence for policies to mitigate such impacts during the response to COVID-19 and during future emergencies. Responding to the disruptions of EHS caused by the COVID-19 pandemic, the IMST/ WHO Regional Office for Europe has developed a comprehensive four-step approach to support countries in restoring, maintaining, and strengthening EHS Delivery and Health System Functions during COVID-19 [10]. The four-step approach consists of a (1) rapid assessment and country situation analysis of the impact of COVID-19 on EHS and health system, (2) development of an Action plan on maintaining EHS during the COVID-19 outbreak, (3) implementation of this Action plan and (4) its monitoring and evaluation. An integral part of the approach is a Survey on the impact of the COVID-19 pandemic on health, well-being and working conditions of health workers.

The aim of this study was to assess the impact of COVID-19 on the health, wellbeing, and working conditions of nurses in Slovakia and to identify gaps in policies to be addressed to increase preparedness of the HW for future emergencies.

## Methods

### Study design and population

A nation-wide, cross-sectional study was conducted among nurses working in health care facilities of all levels and types of care, including social care facilities in Slovakia.

### Data collection

A questionnaire specifically designed for the study was applied. The questionnaire was developed within the WHO Regional Office for Europe to support countries in restoring, maintaining, and strengthening EHS Delivery and Health System Functions during COVID-19 (see supplementary Fig. 1 for details on the approach). In cooperation with the Chamber of Nurses of Slovakia, for the purposes of this study, the original questionnaire was extended with questions on estimation of disruptions of EHS, on considerations to leaving job and its reasons, on the use of telemedicine. Some aspects of the questionnaire were adapted to the national context (E.g., the classification of job titles, or the stratification of health care providers). Before it was applied for this survey in Slovakia, the questionnaire was translated from English to Slovakian language, back translated and pilot tested on a sample of 30 nurses to ensure reliability and validity. After the pilot test, the questionnaire was revised reflecting the feedback from the pilot survey, and then finalized into the form that was used for this study. The internal consistency reliability was assessed using Cronbach’s alpha, yielding a score of 0.74 (95%CI:0.71–0.77) for 13 items in the mental health and wellbeing domain, based on which the used scales can be considered sufficiently reliable [11].

The questions were transformed into an online form and were distributed among nurses as an electronic questionnaire, using the communication channels of the Chamber of Nurses of Slovakia. Emails and social media were used as means of communicating the survey and to ask nurses to participate. No formal selection procedures were applied for respondents, they were enrolled in the survey consecutively. The sample size needed for the study was calculated using expected proportions derived from systematic reviews published prior to this study [8, 9]: for the estimation of the prevalence of COVID-19 infections we have expected the population proportion between 10%-30% which yielded a desired sample size in the 100–400 subjects and for the estimation of mental health symptoms, we have used an estimated population proportion of 25%-40% which yielded a desired sample size in the range of 300–500 subjects (considering the size of the population of nurses in Slovakia being 30,000. Thus, the sample size of 1170 nurses recruited for the study can be considered to be sufficient even when considering the non-random sampling (which was not possible under the circumstances).

The data were collected between November 30^th^ and December 12^th^ of 2021, and the responses refer to the period of January to November 2021.

### Policy identification review

For the purposes of this study, a working group was formed on national level in Slovakia, which was led by experts from the Chamber of Nurses and with members including all authors of this paper based in Slovakia. This group was tasked with identification and review of national policies that are related to management of HW and health emergencies (such as the COVID-19 pandemic). The aim of this activity was to identify pre-existing policies and newly adopted policies that govern HW management during the COVID-19 response and that aim to increase resilience and preparedness of the HW for future emergencies. In the first step, all policies that relate to health emergency management and provision of health care during health emergencies were identified. Subsequently, these were independently studied and analyzed by at least 3 members of the working group to identify sections that relate to HW management. All such sections were noted down and are discussed in this paper in context of improving response to COVID-19 and to increase resilience and preparedness of health systems for future health emergencies.

### Variables and data analysis

Most variables are reported as categorical and are presented as relative frequencies (percentages). Continuous variables are presented either as means with corresponding standard deviations or 95% confidence intervals, or as medians with corresponding interquartile ranges (IQR). Two composite variables were created using multiple single variables as input: 1) the composite score of the impact of workload was calculated as an overall mean of scores assigned to questions on time pressure, burden of responsibility, nervousness, wanting to do something else, fatigue (each was score from 0–3); 2) composite score of mental health and wellbeing was calculated as an overall mean of scores assigned to questions on the frequency of feeling nervous, unable to solve problems, down or depressed, little interest in doing things, unable to control worry, unable to concentrate, or feeling a drop in motivation to work (all scored from 1 to 5).

Respondents were grouped by the type of facility they worked in at the time of the survey to four categories: 1) Hospital—COVID-19 wards, 2) Hospital – non-covid wards, 3) Outpatient or emergency departments (ER) (i.e., general practicioners, specialists and ER), and 4) Other (i.e., social care facilities for the elderly). For all analyses, this stratification was used, in order to assess the differences in the impact of the COVID-19 pandemic on nurses working in different facilities. The R statistical software was used for all analyses [12]. The Kolmogorov–Smirnov and Shapiro tests were used to test for the normality of distribution of continuous variables. The One-way ANOVA test was used to test for differences in means, Kruskal–Wallis test to test for differences in medians, and the Chi-squared test was used to test for the significance of the differences between categorical variables. P < 0.05 was considered statistically significant.

### Ethical consideration

The study has been approved by the Ethical committee of the Faculty of Health Sciences and Social Work of the Trnava University on November 15^th^ 2021.

## Results

Overall, 1170 nurses participated in the survey. The distribution of the respondents into facility groups was relatively even: most of them worked at non-covid hospital wards (30%), followed by outpatients and emergency care facilities (29%), COVID-19 hospital wards (24%), and about 20% worked in other facilities (social care facilities for the elderly). The mean age of the participating nurses was 43.9 years (SD = 10.7), the oldest being nurses in the Other group of facilities (47.4, SD = 8.9), and the youngest being the nurses in COVID-19 hospital wards (40.9, SD-11). Most of the respondents (1121, 96%) were females.

### Direct impact of COVID-19 on health of nurses

Table 1 shows the patterns and extent of the direct impact of COVID-19 on the health workers in our survey. About a third of the respondents (32% overall) tested positive for COVID-19, with this proportion being the highest in the Other group (35%) and lowest in regular hospital wards (29%). About 3% of respondents reported testing positive two or more times. Most of those reporting being infected with COVID-19 developed mild disease (54%), the highest proportion of those with severe disease were at COVID-19 wards (6%), the lowest in regular hospital wards (1%). Almost two thirds reported long-covid symptoms, underlining the long-term nature of the consequences of COVID-19 on the HW. The details of the distribution of symptom prevalence are presented in Table 1: the medium of the number of reported long-covid symptoms was 4 (IQR 2–6), with the three most reported being extreme tiredness (58% or respondents), joint pain (53%) and loss of appetite (49%). Most of those reporting to test positive for COVID-19 were non vaccinated (63%) or were vaccinated with one dose of vaccine (9%). In general, 82% of all respondents were fully vaccinated, with about 13% reporting that they do not plan to get the vaccine.

**Table 1:** COVID-19 related health status of nurses in Slovakia by type of health care provider during the COVID-19 pandemic

Variable	Hospital (COVID-19 ward)	Hospital (Regular Ward)	Outpatient or ER	Other	Total	*P* Value
**Age (mean, 95% CI)**	40 (39.7–42.3)	41.8 (40.5–43)	46.4 (45.4–47.3)	47.4 (46.2–48.7)	43.9 (43.3–44.5)	< 0.001
**COVID-19 Vaccination Status (N, %)**						0.053
Yes, full	223 (81%)	265 (77%)	289 (87%)	158 (83%)	935 (82%)	
Yes, one dose	11 (4%)	7 (2%)	7 (2%)	4 (2%)	29 (3%)	
No, but planning	7 (3%)	16 (5%)	5 (2%)	7 (4%)	35 (3%)	
No, not planning	36 (13%)	55 (16%)	32 (10%)	22 (12%)	145 (13%)	
**Tested Positive for COVID-19 (N, %)**						
Once	91 (33%)	103 (29%)	104 (31%)	69 (35%)	367 (32%)	0.594
Two or more times	8 (3%)	6 (2%)	8 (2%)	7 (4%)	29 (3%)	
**When positive for COVID-19 (N, %)**						< 0.001
Quarantined	91 (96%)	103 (95%)	99 (93%)	63 (84%)	356 (93%)	
Admitted to hospital	1 (1%)	3 (3%)	4 (4%)	1 (1%)	9 (2%)	
Worked while positive	3 (3%)	2 (2%)	4 (4%)	11 (15%)	20 (5%)	
**COVID-19 Disease severity (N, %)**						< 0.001
Mild	61 (64%)	63 (58%)	44 (41%)	41 (55%)	209 (54%)	
Moderate	28 (30%)	45 (41%)	59 (55%)	32 (43%)	164 (42%)	
Severe	6 (6%)	1 (1%)	5 (5%)	2 (3%)	14 (4%)	
**Vaccinated when positive COVID-19 (N, %)**						0.401
Ful vaccination	19 (20%)	30 (28%)	34 (31%)	26 (35%)	109 (28%)	
One dose	11 (12%)	8 (7%)	10 (9%)	5 (7%)	34 (9%)	
Not vaccinated	65 (68%)	71 (65%)	65 (60%)	44 (59%)	245 (63%)	
**Presence of Long-Covid symptoms (N, % Yes)**	70 (56%)	98 (66%)	77 (63%)	49 (53%)	294 (60%)	0.370
**Long-Covid symptoms present (N, %)**						
Extreme tiredness (fatigue)	42 (61%)	59 (61%)	38 (50%)	29 (59%)	168 (58%)	0.464
Shortness of breath	24 (35%)	34 (35%)	28 (37%)	19 (39%)	105 (36%)	0.965
Chest pain or tightness	14 (20%)	23 (24%)	12 (16%)	13 (27%)	62 (21%)	0.465
Problems with memory and concentration ("brain fog")	24 (35%)	26 (27%)	22 (29%)	18 (37%)	90 (31%)	0.539
Difficulty sleeping (insomnia)	24 (35%)	28 (29%)	26 (34%)	20 (41%)	98 (34%)	0.537
Heart palpitations	23 (33%)	30 (31%)	21 (28%)	15 (31%)	89 (31%)	0.905
Depression and anxiety	13 (19%)	13 (13%)	10 (13%)	11 (22%)	47 (16%)	0.415
Joint pain	40 (58%)	51 (53%)	38 (50%)	25 (51%)	154 (53%)	0.791
Tinnitus	10 (15%)	10 (10%)	8 (10%)	8 (11%)	9 (18%)	0.373
Eating disorders	9 (13%)	22 (23%)	10 (13%)	13 (27%)	54 (19%)	0.111
Rash	7 (10%)	12 (12%)	7 (9%)	7 (14%)	33 (11%)	0.809
High temperature	10 (15%)	20 (21%)	11 (15%)	15 (31%)	56 (19%)	0.098
Cough, sore throat	13 (19%)	36 (37%)	20 (26%)	23 (47%)	92 (32%)	< 0.01
Loss of taste	26 (38%)	36 (37%)	29 (38%)	23 (47%)	114 (39%)	0.679
Loss of appetite	30 (44%)	44 (45%)	41 (54%)	28 (57%)	143 (49%)	0.334
**Number of Long-Covid symptoms (median, IQR)**	4 (2–7)	4 (3–6)	3.5 (2–6)	5 (3–8)	4 (2–6)	0.272

### Workload, working conditions and mental health

**Fig. 1:**
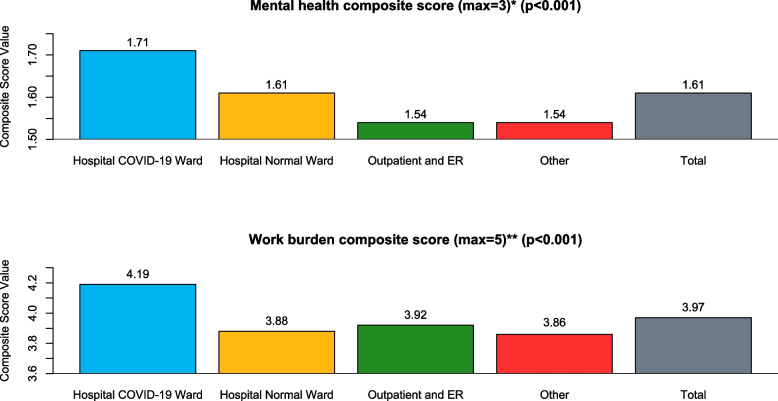
Composite scores of the impact of the COVID-19 on mental health and the perceived work burden in nurses during the COVID-19 pandemic in Slovakia by type of health care provider. * Composite score calculated as a mean of scores of four indicators of mental health impact: nervousness or irritation, inability to solve problems, being depressed or down, no interest in doing things, inability to stop worrying, inability to stay focused, and lack of motivation; each item scored on a scale from 0–3 (3 = almost every day, 0 = not at all). **Composite score calculated as a mean of scores of four indicators of work burden: feeling time pressured, feeling the burden of responsibility, feeling nervous or irritated, wanting to do something else, or feeling fatigue after a few hours of work; each item scored on a scale from 1–4 (5 = fully agree, 1 = fully disagree)

Table 2 reports on workload, working conditions and their consequence on mental health and well-being among the responding nurses. About 70% of nurses reported feeling time pressure, 80% felt high burden due to job responsibilities, 61% felt increasingly nervous or irritated after a few hours of work, more than half (58%) felt like doing something else and over two thirds (68%) felt fatigue or weakness after a few hours of work. The composite score of the impact of workload combining all the above-mentioned consequences showed a median score of 2.8 (i.e., 56% out of a maximum possible 5 points), highest in nurses working in covid wards (median score of 3); see Fig. 1.

**Table 2:** Impact of the COVID-19 pandemic on mental health and well-being and working conditions of nurses in Slovakia by type of health care provider during the COVID-19 pandemic

Variable	Hospital (COVID-19 ward)	Hospital (Regular Ward)	Outpatient or ER	Other	Total	*P* Value
**Work related burden symptoms (N, %)**						
Often feeling time pressure during work	231 (83%)	231 (66%)	216 (65%)	127 (65%)	805 (70%)	< 0.001
High burden due to responsibility	241 (86%)	263 (75%)	261 (78%)	158 (81%)	923 (80%)	< 0.01
Feeling nervous or irritated after few hours of work	192 (69%)	206 (59%)	200 (60%)	111 (58%)	709 (61%)	0.037
Wanting to do something else after few hours of work	180 (64%)	203 (58%)	186 (56%)	104 (54%)	673 (58%)	0.081
Feeling fatigue/weakness after few hours of work	211 (75%)	222 (64%)	228 (68%)	124 (64%)	785 (68%)	< 0.01
**Composite work burden score (mean, 95%CI)**	3 (2.9–3.1)	2.8 (2.7–2.9)	2.8 (2.7–2.9)	2.8 (2.7–2.9)	2.8 (2.8–2.9)	< 0.001
**Did you think of leaving your job (N, % Yes)**	212 (76%)	253 (73%)	221 (66%)	122 (62%)	808 (70%)	< 0.01
**Reasons for thinking of leaving (N, %)**						
Inadequate pay	62 (29%)	99 (39%)	79 (36%)	40 (33%)	280 (35%)	
Lack of appreciation	33 (16%)	46 (18%)	42 (19%)	19 (16%)	140 (17%)	
Lack of personnel	26 (12%)	27 (11%)	5 (2%)	10 (8%)	68 (8%)	< 0.01
Work stress	89 (42%)	78 (31%)	94 (43%)	52 (43%)	313 (39%)	
Lack of equipment	2 (1%)	3 (1%)	1 (1%)	1 (1%)	7 (1%)	
**How often have you felt (N, %)**						
Nervous or irritable	165 (64%)	191 (59%)	179 (59%)	89 (51%)	624 (59%)	0.062
Unable to solve my problems	97 (41%)	108 (38%)	104 (38%)	46 (30%)	355 (38%)	0.179
Down, depressed or hopeless	125 (48%)	125 (39%)	113 (37%)	57 (33%)	420 (40%)	< 0.01
Little interest or pleasure in doing things	120 (46%)	133 (41%)	128 (44%)	63 (36%)	444 (42%)	0.229
Unable to control worrying	123 (48%)	149 (49%)	134 (46%)	75 (43%)	481 (47%)	0.659
Problems with concentration	93 (38%)	108 (34%)	95 (33%)	56 (31%)	352 (34%)	0.469
Drop in work motivation	153 (58%)	183 (56%)	138 (46%)	81 (45%)	555 (52%)	< 0.01
**Composite mental health score (mean, 95%CI)**	1.7 (1.6–1.8)	1.6 (1.5–1.7)	1.5 (1.4–1.6)	1.5 (1.4–1.7)	1.6 (1.5–1.7)	0.073
**Availability of protective equipment (N, % Yes)**	94 (34%)	150 (43%)	112 (34%)	73 (38%)	429 (37%)	0.04
**Situation critical due to lack of nurses (N, %)**						
Yes, to a large extent	157 (56%)	177 (51%)	53 (17%)	82 (43%)	469 (41%)	< 0.001
Yes, partially	105 (38%)	140 (40%)	125 (40%)	78 (41%)	448 (40%)	
**Situation critical due to lack of physicians (N, %)**						
Yes, to a large extent	30 (11%)	38 (11%)	32 (10%)	22 (12%)	122 (11%)	< 0.001
Yes, partially	116 (42%)	144 (42%)	113 (36%)	55 (31%)	428 (38%)	
**Situation critical due to lack of equipment (N, %)**						
Yes, to a large extent	126 (46%)	70 (21%)	29 (13%)	16 (9%)	241 (24%)	< 0.001
Yes, partially	94 (34%)	112 (33%)	38 (16%)	31 (18%)	275 (27%)

Furthermore, 59% of the respondents reported feeling nervous or irritable, 52% reported a drop in work motivation, 47% reported not being able to stop worrying and 42% reported feeling like having little interest or pleasure doing things for more than half of days. Feeling unable to solve problems, feeling down, depressed, or hopeless, and having problems with concentration were all reported to be present for more than half of days in over a third of respondents. The composite score of mental health and wellbeing combining the above impacts shows an average of 1.6 (i.e., 53% out of a maximum of 3 points); consult Fig. 1 for details.

All surveyed mental health and wellbeing impacts were reported in the highest extent by nurses working in COVID-19 hospital wards, suggesting that nurses working in these facilities have been at significantly higher risk for mental health problems.

In all the facility groups, respondents reported that in the facility they work the situation was critical at some point due to lack of nurses: reported by 81% of respondents in general, in over 90% in hospital facilities. Similarly (although to a lesser extent), many respondents reported that the situation was critical due to lack of physicians (49%), and about 51% reported that the situation was critical due to lack of equipment (this was reported to a higher extent in hospitals). Protective equipment has been fully available in about 37% of facilities overall.

Overall, 70% of the respondents admitted that they were thinking about leaving their job – the proportion of such respondents was significantly higher in hospitals (73%-76%), compared to other facilities (62%-66%); extreme workload was given most often as the reason (39%), along with inadequate pay (35%), or lack of appreciation (17%).

### Disruptions of health care and use of telemedicine

Table 3 shows an overview of service disruptions due to COVID-19 in the facilities where the respondents were employed. A disruption of preventative care, therapeutic care, or long-term care was reported to be higher than 50%, compared to pre-covid times was reported by about a third of respondents. The detailed structure of these disruptions is presented in Table 3 and in Supplementary Fig. 2. About 60% of the respondents reported that they used telemedicine, emails, and phones for patient consultations in a higher or much higher extent than before the pandemic; this was especially true for outpatients. Also, e-prescriptions were used significantly more often than before the pandemic in most facilities. However, about 64% of the respondents reported that they were not specifically trained in using telemedicine to provide care. Supplementary Fig. 3 shows the patterns of specific use of telemedicine in the different facility groups: use for teleconsultations was reported by nurses working in outpatient facilities (75%) and other facilities (68%) significantly more often than in hospitals (45%-48%); telemonitoring was reported mainly to be used in hospitals (18% in COVID-19 wards and 12% in general wards); to a lesser extent was telemedicine used for tele-triage in all facilities.

**Table 3:** Impact of the COVID-19 pandemic on disruptions of health care and the use of telehealth during the COVID-19 pandemic in Slovakia by type of health care provider from the perspective of nurses

Variable	Hospital (COVID-19 ward)	Hospital (Regular Ward)	Outpatient or ER	Other	Total	*P* Value
**Disruption of health care (N, % Yes)**	206 (77%)	228 (67%)	157 (47%)	100 (53%)	691 (61%)	< 0.001
**Level of disruption of preventive care (N, %)**						
< 25%	30 (15%)	60 (27%)	66 (42%)	29 (29%)	185 (27%)	
26–50%	89 (43%)	97 (43%)	67 (43%)	36 (36%)	289 (42%)	< 0.001
51–75%	63 (31%)	47 (21%)	14 (9%)	24 (24%)	148 (22%)	
75% or more	23 (11%)	22 (10%)	9 (6%)	10 (10%)	64 (9%)	
**Level of disruption of therapeutic care (N, %)**						
< 25%	34 (17%)	64 (28%)	74 (48%)	34 (35%)	206 (30%)	
26–50%	85 (42%)	95 (42%)	60 (39%)	36 (37%)	276 (41%)	< 0.001
51–75%	59 (29%)	50 (22%)	11 (7%)	23 (24%)	143 (21%)	
75% or more	25 (12%)	18 (8%)	8 (5%)	5 (5%)	56 (8%)	
**Level of disruption of rehabilitation care (N, %)**						
< 25%	56 (28%)	78 (36%)	52 (44%)	40 (43%)	223 (36%)	
26–50%	58 (29%)	64 (29%)	38 (32%)	24 (26%)	184 (29%)	0.029
51–75%	44 (22%)	48 (22%)	18 (15%)	14 (15%)	124 (20%)	
75% or more	40 (20%)	28 (13%)	10 (9%)	15 (16%)	93 (15%)	
**Use of phone to provide consultations (N, % Yes)**						
Not used	105 (41%)	164 (52%)	39 (12%)	50 (27%)	358 (33%)	
Used about the same as before	28 (11%)	38 (12%)	14 (4%)	15 (8%)	95 (9%)	< 0.001
Use slightly more than before	56 (22%)	54 (17%)	46 (14%)	50 (27%)	206 (19%)	
Used much more than before	65 (26%)	57 (18%)	230 (70%)	69 (38%)	421 (39%)	
**Use of email to provide consultations (N, % Yes)**						
Not used	194 (75%)	240 (75%)	92 (28%)	90 (50%)	616 (57%)	
Used about the same as before	17 (7%)	22 (7%)	29 (9%)	17 (9%)	85 (8%)	< 0.001
Use slightly more than before	21 (8%)	29 (9%)	46 (14%)	30 (17%)	126 (12%)	
Used much more than before	26 (10%)	31 (10%)	162 (49%)	44 (24%)	263 (24%)	
**Use of e-prescriptions (N, % Yes)**						
Not used	135 (63%)	170 (58%)	28 (9%)	73 (42%)	406 (40%)	
Used about the same as before	16 (8%)	32 (11%)	40 (12%)	27 (16%)	115 (11%)	
Use slightly more than before	26 (12%)	34 (12%)	38 (12%)	20 (12%)	118 (12%)	< 0.001
Used much more than before	36 (17%)	56 (19%)	225 (68%)	52 (30%)	369 (37%)	
**Feel trained for use of telemedicine (N, %)**						
Yes, fully	25 (11%)	18 (6%)	46 (17%)	17 (11%)	106 (11%)	
Yes, partially	69 (29%)	50 (17%)	79 (30%)	40 (26%)	238 (25%)	< 0.001
Not trained	141 (60%)	230 (77%)	141 (53%)	99 (64%)	611 (64%)	

### Policies revised or adopted for the response to COVID-19

Since the start of the COVID-19 pandemic, policies in Slovakia were adopted, or existing policies were revised in order to provide effective response to COVID-19 and to maintain EHS. Policies that are aimed at the HW include standard operating procedures (SOP) for providing health care during the COVID-19 pandemic, guidance for testing the HWs for SARS-CoV-2, guidance for ensuring IPC in health care facilities, guidance for claiming sickness benefits after contracting COVID-19 or close contact with persons testing positive for SARS-CoV-2, methodological guidance for SARS-CoV-2 testing, or professional guidance for establishment of testing and vaccination centers.

On the level of health care facilities, internal guidance and managerial decisions were adopted for the implementation of patient triage, designation of wards, shortening of operation hours of some parts of health facilities, or supply of PPEs. On national level, the National Institute of Public Health, the working group for management of crises of the Ministry of Health and the chief epidemiologist of Slovakia developed a National pandemic plan for COVID-19 which was adopted in August of 2022. While the above-mentioned policies include in a direct or indirect way strategies related to HW management, no specific standalone strategy or policy has been adopted on national level for this purpose. This study clearly shows the impact of COVID-19 on the HW and the long-term implications for the available capacities for future emergencies. Thus, a specific policy on supporting the development and maintain of the HW is warranted to increased preparedness and resilience of the health systems for future emergencies.

## Discussion

### Main findings

A nation-wide survey on a sample of 1170 nurses working in various health care facilities on different levels of health care in Slovakia in 2021 was conducted in order to assess the impact of the COVID-19 pandemic on their health, working conditions and mental wellbeing, to provide an overview of policies adopted for the mitigation of these impacts and for the management of the HW during the COVID-19 response, and to identify gaps in policies to be filled in to increase the resilience and preparedness of the heath system for future emergencies.

About a third of the respondents tested positive for COVID-19 by November of 2021, most of them developing mild disease. Almost two thirds reported long-covid symptoms (medium of the number of reported symptoms was 4). About 13% of respondents reported that they do not plan to get the vaccine. The composite score of the impact of workload showed a median score of 2.8 (i.e., 56% of the maximum 5 points), while the median composite score of mental health and wellbeing was 1.9 (i.e., 63% of a maximum of 3 points). All surveyed mental health and wellbeing impacts were reported to a higher extent by nurses working in COVID-19 hospital wards (as compared to other groups), suggesting that nurses working in these facilities have been at significantly higher risk for mental health problems. Significant disruptions of preventive, therapeutic and rehabilitation care were reported, with relatively high use of telemedicine technologies to mitigate them. Overall, about 70% of the respondents thought of leaving their job, in most cases due to working stress or inadequate pay. While policies to protect and support the mental health and wellbeing along with guidelines to lower the exposure and limit the risk of COVID-19 among health workers were adopted to aid the response and maintain EHS, specific longer-term policies to maintain and protect the HW for better resilience and preparedness for future emergencies were lacking.

### Comparison with literature

Health workers have been previously identified as being under increased risk of COVID-19, as compared to the general population: a global seroprevalence study estimated that the seroprevalence was about 4.9% overall, with health workers showing substantially higher values [6], while another review of population-based studies showed seroprevalences ranging from 0.42% to 13.6% [13]. Specifically in the population of nurses, a systematic review of studies relating mostly to 2020 estimated the pooled seroprevalence of SARS-CoV-2 antibodies to be at 8.1% overall, and 10.3% in studies from Europe. The authors further conclude, that this comparable to other HW [14]. Another systematic review of studies produced an estimate of 8% and identified male health workers and ethnic minority HW to be at higher risk of infection [15], and a review looking at an overall population of about 173,000 health workers estimated the overall seroprevalence at 8.6% and at 7.7% in Europe [16]. A review looking at studies from the beginning of the pandemic until September of 2020 reported a seroprevalence among the HW ranging from 0.7% to 45.3% [17]. In this study, 32% of nurses reported to have tested positive for SARS-CoV-2 by the end of November of 2021. The relatively higher incidence can be explained by the fact that we were analyzing the period of January to November 2021, while most of the published studies were looking at data from 2020. This study also found that there are differences in the reported incidence of positive tests between nurses working in facilities with different levels of exposures to infections – those working in COVID-19 hospital wards and social care facilities were at higher risk compared to outpatients, ER and regular hospital wards. This is consistent with a finding from systematic review which showed that health workers in contact with infected persons had higher risk compared to health workers without known contact (the estimated risk was 2.1 time higher) [6].

The impact of the COVID-19 pandemic on the mental health and wellbeing of health workers has also been previously studied. A review of 38 studies estimated the pooled prevalence of mental health problems for post-traumatic stress disorder, anxiety, depression, and distress in health workers at 49%, 40%, 37% and 37%, respectively [18]. Another summary analysis produced an estimation of the prevalence of anxiety, depression, stress, and insomnia at 26.3%, 25.9%, 26.2%, and 31.3%, respectively [19]. A review of 22 studies estimated the prevalence of depression symptoms at 22% and that of insomnia symptoms at 57% [20]. In this study, depression symptoms were reported by 40% of the respondents, and the composite mental health impact score showed a mean score of 1.6 which is 53% of the maximum score. Thus, the findings of our study are consistent with the published literature. This study also revealed that nurses working in COVID-19 wards (i.e., having the highest workload and work stress related to treating COVID-19 patients) report significantly higher prevalence of mental health symptoms. This is also confirmed by published literature, where the prevalence of anxiety, depression, stress, and insomnia were found to the highest extent among frontline HW as compared to general health workers and the general population [19].

The impacts of the COVID-19 pandemic on the psychological well-being of the HW is substantial on global level, and governments and managements have key responsibilities to protect and preserve the mental health of the HW [21]. A review of 16 studies focusing on implementation of an intervention aimed at supporting the resilience or mental health of frontline workers during disease outbreaks found that such interventions included workplace interventions (e.g., training, structure, and communication), psychological support interventions (E.g., counselling and psychology services and multifaceted interventions. However, they conclude that there is a lack of evidence that could inform the selection of interventions that are beneficial to the resilience and mental health of frontline HW [22]. Thus, it is important that studies provide evidence on the benefit of specific interventions and share good practices with their implementations, so that policies and strategies to increase resilience can be better planned.

In general, the mental health of the HW should be prioritized for both long-term occupational capacity and short-term crisis response, with the following strategies to be considered by managers: assess and minimize additional COVID-19-related occupational psychosocial risks for stress; ensuring access to psychosocial support along with promotion of health seeking; organize schedules to include breaks, minimize other work-related stress and activate peer support; train health leads in basic psychosocial skills [3, 4]. In Slovakia, triggered by the impact of the COVID-19 pandemic on mental health, a governmental working group of experts working in the field of mental health was formed and tasked with conceptualization of the Governmental committee for mental health; the committee was established in February of 2021 to advise the government in tackling mental health related problems in the population and in specific sectors [23]. As one of the first initiatives, psychosocial help lines were launched for the public and for the HW to support people and HW in need of such help; this was accompanied with a media campaign [24].

One other major consequence of the increased workload during the COVID-19 identified by this survey was the intention to leave the job. The survey found that 70% of the respondents considered leaving their job, and the main reason given was the increased workload and stress. Indeed, the Slovak Chamber of Nurses and Midwives reported a 20% increase in nurses leaving their jobs during the study period, when compared to the same period in 2020 [25]. While it is not clear whether this trend can be entirely attributed to the COVID-19 pandemic, such findings indicate that the consequences for the HW, and health systems of countries in general, and for their resilience and preparedness for future emergencies may be substantial. The scientific literature on this topic is relatively scarce, but a survey of the American Association of Critical-Care Nurses on over 6,500 critical care nurses found that 66% have considered leaving nursing after their experience in the pandemic [26], which supports our findings.

Even before the COVID-19 pandemic, the number of registered nurses was lower (5.7 nurses per 1000 inhabitants), when compared to some similar countries, like the Czech Republic (8.1 per 1000). This suggest that the HW is understaffed, which makes the health system less resilient for emergencies. In response, the Slovak Chamber of Nurses and Midwives outlined a plan to improve the situation of nurses in the country, which includes nine strategies: increase wages; improve working conditions; enforcing minimal requirements for staffing of health facilities; improving the management of nursing care; introducing a credit based lifelong learning program; introducing additional benefits; increasing motivation for nursing studies; supporting the professional education of nurses; and building teams in nursing care [25]. It is crucial, that such a plan is implemented as it has a potential to strengthen the HW in general and increase preparedness of the health system for emergencies.

Telemedicine technologies were reported to be used and implemented in a significantly higher extent, compared to the period before the COVID-19 pandemic in our study, which is consistent with the lessons learned during the pandemic in various countries with different levels of health system. While telemedicine has already been increasingly introduced for health services delivery, the start of the COVID-19 pandemic has substantially accelerated its use: for example a study on over 6 million employer health plan beneficiaries estimated more than a 20-fold increase in use of telemedicine after March 13 2020, compared to the previous year [27], another study on a national sample in the US estimated that around half of the surveyed patients used TH for consultation as early as in May 2020 [28]. Published research evidence showed that during the outbreak, technologies including remote services in healthcare system were successfully implemented upon lockdowns in periods of high transmission rates [29]. The areas of implementation of such technologies included webinars to the healthcare staff, internship learning, medical consultations in non-surgical healthcare services, or others. General practitioners, allergy physicians, endocrinologists, dental specialists, and many others switched their practice to expending telemedicine response, giving more answers by phone, email and video consultations [30]. Some areas may implement telemedicine as a complete substitute for face-to face consultations, while in others it may be used for prioritizing patients by remote consultations to face-to-face treatment, imaging diagnostic, dropping of patients from treatment [31].This is confirmed in this study, where the use of telemedicine has increased significantly during the COVID-19 pandemic, when compared to periods before the pandemic. This is partly the result of the strategical decision of the Ministry of health to limit personal visits with patients to those in need of acute care and provide nursing care remotely in chronically ill patients or those without acute need of care. This has been published as a guidance by the Ministry of health of Slovakia in March 2020. Similar guidance was developed and published on national level for general practitioners [32]. Thus, while disruptions of health care have been reported in our survey on all levels of care, timely policies, and decisions to implement telemedicine helped to overcome them.

### Bias and limitations

This study presents self-reported data, which may impose information bias to the results. However, we note that there are several factors that conform the representativeness and generalizability of our findings. First, the fact that the respondents represent 69 out of the total of 79 districts in Slovakia, and that the mean age of respondents in this survey (44 years) is similar to the nation-wide mean age of nurses as published by the Slovak Chamber of Nurses and Midwives (46 years) suggest that the distribution of our respondents represents the nurses well. Second, the sample size of 1170 is higher than the calculated sample size needed for the study and provides sufficient power to generalize the findings to the whole population of about 30,000 registered nurses in Slovakia. Thus, despite the above limitations, this study provides valuable and valid data on the impact of COVID-19 on health and working conditions of nurses in Slovakia. We have not had the means to confirm the presence of COVID-19 infection among those nurses who reported such infections. While this may pose bias, we expected that all reported cases were confirmed by a PCR test, as per protocols being in place at the time of the study in Slovakia. Thus, we expect the presented self-reported prevalence of COVID-19 infections to reflect the true prevalence among the nurses.

It can be argued that conducting country-level cross-sectional studies may not be beneficial or justified after systematic reviews were published on topic. However, we argue that systematic reviews which we refer to in this paper summarize studies conducted in different countries, with different study design, different populations, different time periods, in different types of health care facilities and different numbers of centers included, and in general differing in study design and setting. Thus, a large inherent variability is introduced to such reviews, which may bias the findings and, in our opinion, do not provide sufficiently valid results that could be generalizable to specific countries and settings and do not provide evidence for policy makers to act upon that would be sufficiently specific and detailed. Our study was designed to provide a holistic overview of the impact of the COVID-19 pandemic on nurses in Slovakia, that included their physical and mental health but also their working conditions and overall work burden. We provide a stratified comparison between nurses working in different wards and being under different risk of infection or other impacts. Furthermore, we provide an analysis of policies being in place and bring all these in context with each other. Thus, we believe that our study provides a much more valid set of findings that could be used to improve preparedness and resilience of health system in Slovakia for future health emergencies. We believe this provides sufficient justification of our study and its added value, compared to existing systematic reviews.

## Conclusions

Our study showed that the COVID-19 pandemic poses a substantial burden on the health, wellbeing and working conditions of nurses in Slovakia and that a large proportion of nurses considered leaving their jobs because of work overload or low salaries. Human resource strategies should be adopted to attract, retain and continuously invest in HW development including in emergency preparedness and response. Such an approach may improve the resilience and preparedness of the health system in Slovakia for future emergencies.

## Supplementary Information

**Additional file 1:** **Supplementary Figure 1.** Outline of the sequence of steps and context of the Four step approach to maintain, restore and strengthen the provision of EHS during the COVID-19 and to increase the preparedness and resilience of health systems for future emergencies. **Supplementary Figure 2.** Levels of disruption of health care due to the COVID-19 pandemic in Slovakia by type of health care provider from the perspective of nurses*. **Supplementary Figure 3.** Patterns of use of telemedicine for specific purposes during the COVID-19 pandemic in Slovakia by type of health care provider.

## Data Availability

The datasets generated and/or analysed during the current study are available upon reasonable request from the authors, please contact Marek Majdan at mmajdan@truni.sk.

## Abbreviations

HW: Health Workforce
EHS: Essential Health Services
WHO: World Health Organization
PPE: Personal Protective Equipment
SARS: Severe Acute Respiratory Syndrome
IQR: Interquartile Range
SD: Standard Deviation
