# Supplementary material for: The impact of the COVID-19 pandemic on the health and working conditions of nurses and its implications for policies: a cross-sectional study in Slovakia
Source: BMC Nurs. 2023 May 29;22:185. doi: 10.1186/s12912-023-01356-z (PMC10226785; doi:10.1186/s12912-023-01356-z)

The Impact of the COVID-19 Pandemic on the Health and Working Conditions of Nurses and its Implications for Policies: A Cross-sectional Study in Slovakia

Supplemental Material

Supplementary Figure 1: Outline of the sequence of steps and context of the Four step approach to maintain, restore and strengthen the provision of EHS during the COVID-19 and to increase the preparedness and resilience of health systems for future emergencies

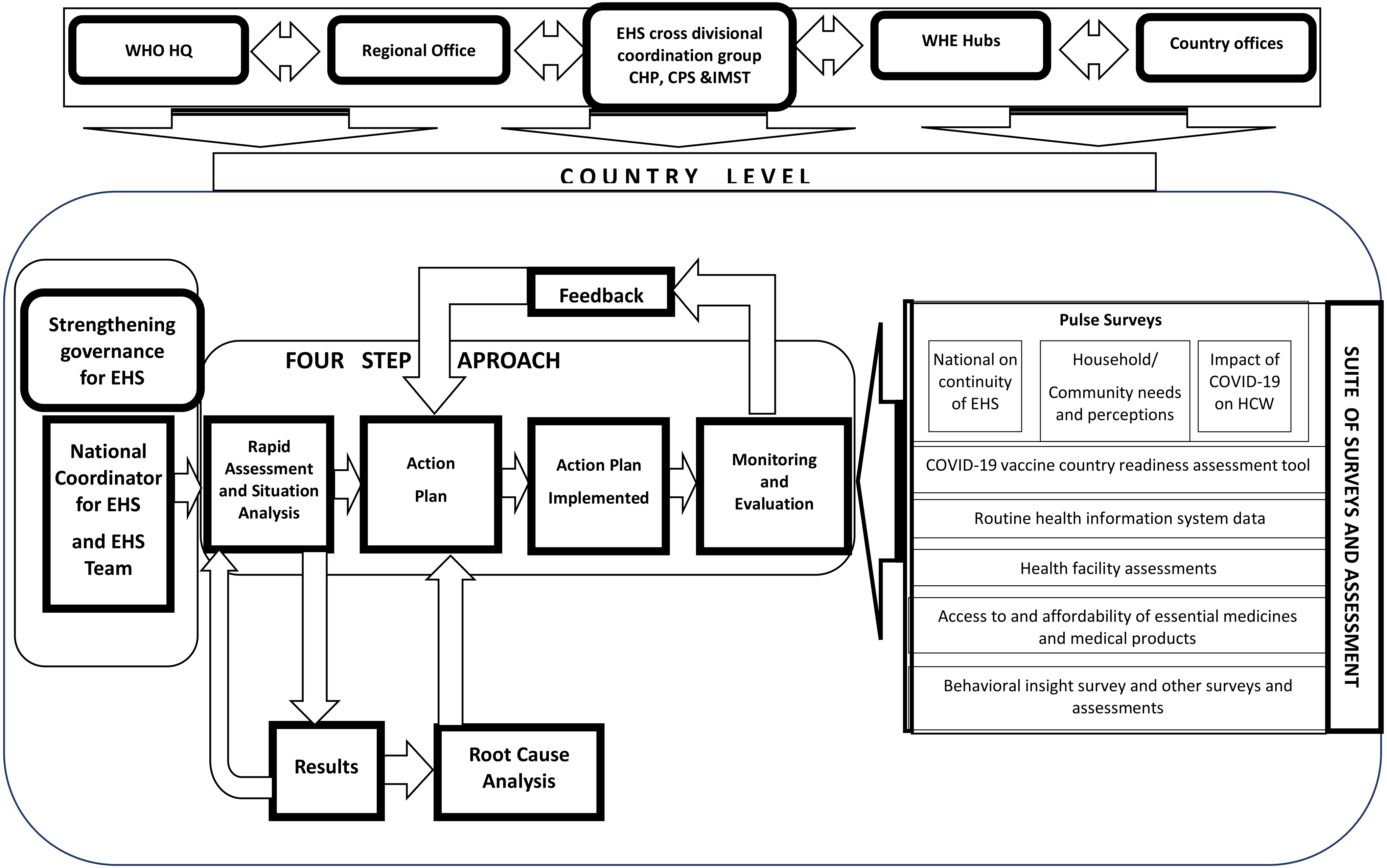

Supplementary Figure 2: Levels of disruption of health care due to the COVID-19 pandemic in Slovakia by type of health care provider from the perspective of nurses*

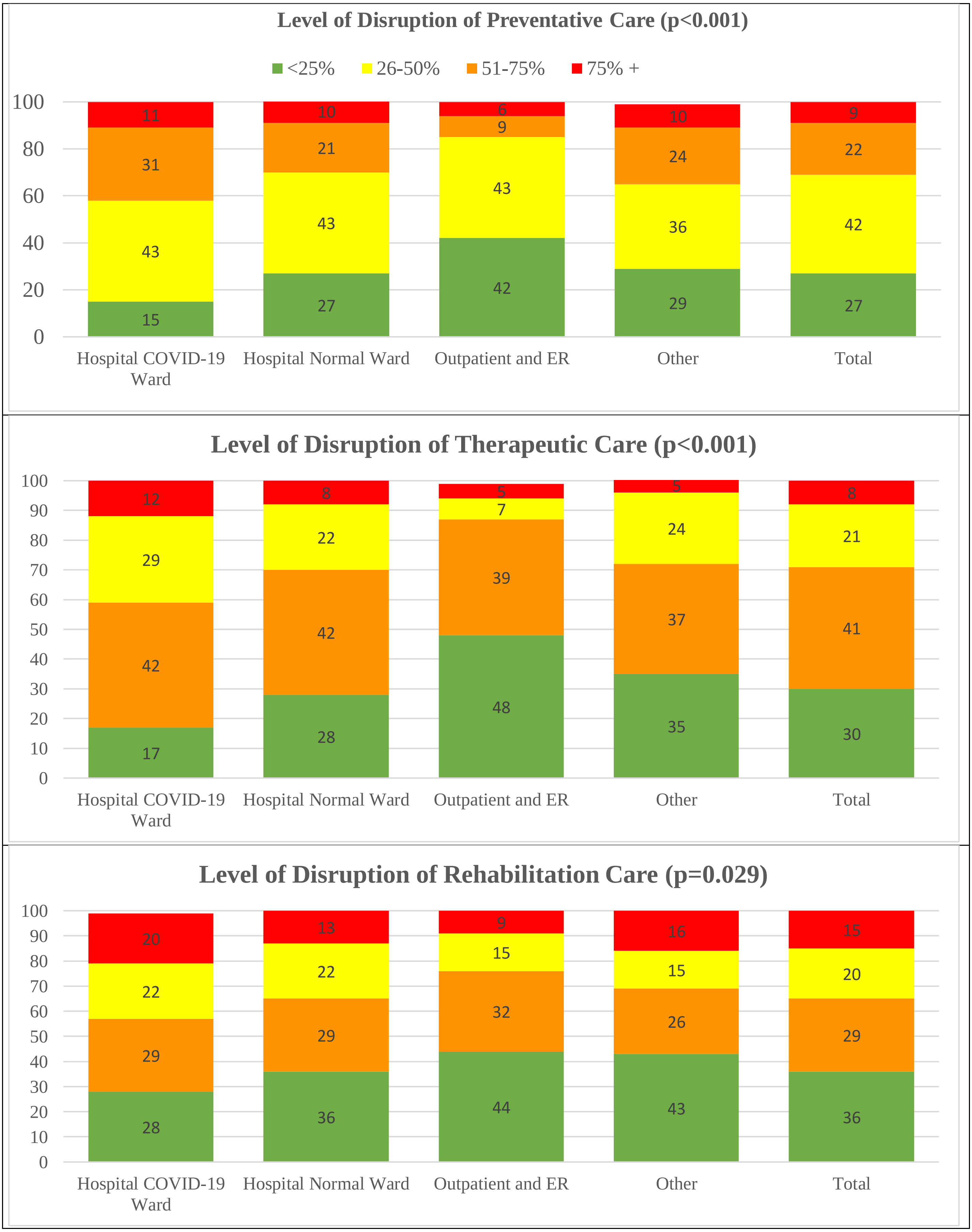

The level of disruption is presented as four categories (<25%, 26-50%, 51-75%, 75+%); the graphs show the percentage of responding nurses estimating the disruption of care at the facility they work at one of the four levels (separately for preventative, therapeutic and rehabilitation care)

Supplementary Figure 3: Patterns of use of telemedicine for specific purposes during the COVID-19 pandemic in Slovakia by type of health care provider

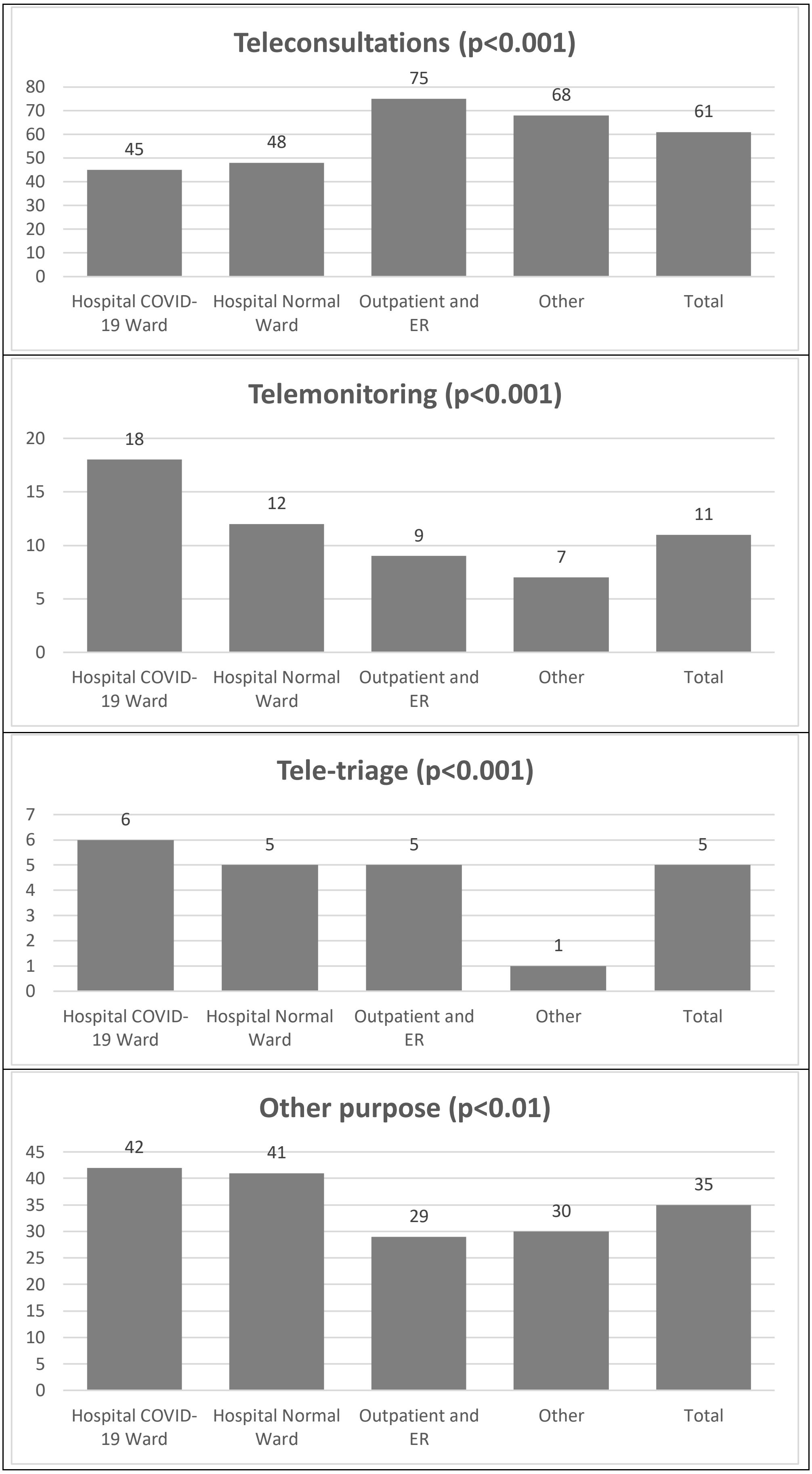

Supplement: Supplementary file 1 — Additional file 1: Supplementary Figure 1. Outline of the sequence of steps and context of the Four step approach to maintain, restore and strengthen the provision of EHS during the COVID-19 and to increase the preparedness and resilience of health systems for future emergencies. Supplementary Figure 2. Levels of disruption of health care due to the COVID-19 pandemic in Slovakia by type of health care provider from the perspective of nurses*. Supplementary Figure 3. Patterns of use of telemedicine for specific purposes during the COVID-19 pandemic in Slovakia by type of health care provider. [file 12912_2023_1356_MOESM1_ESM.pdf]
